# Psychological Well-Being and Life Satisfaction in Children and Adolescents with Chronic Illness: The Role of Depression, Nonproductive Thoughts, and Problematic Internet Use

**DOI:** 10.3390/children12050657

**Published:** 2025-05-21

**Authors:** Karolina Eszter Kovács, Péter Boris, Beáta Erika Nagy

**Affiliations:** 1 Institute of Psychology, Faculty of Arts, University of Debrecen, 4032 Debrecen, Hungary; 2 Laki Kálmán Doctoral School, University of Debrecen, 4032 Debrecen, Hungary; boris.peter@med.unideb.hu; 3 Pediatric Psychology and Psychosomatic Unit, Pediatric Rehabilitation, Institute of Pediatrics, Faculty of Medicine, University of Debrecen, 4032 Debrecen, Hungary; nagy.beata@med.unideb.hu

**Keywords:** illness burden, depression, nonproductive thoughts, well-being, illness representation

## Abstract

Theoretical background: The study of psychological well-being in children and adolescents living with chronic illness is of particular relevance, as the physical and psychosocial aspects of the illness can have a significant impact on their quality of life. Previous research has highlighted that depression, nonproductive thoughts and various aspects of problematic internet use may be related to life satisfaction and ways of coping with illness. This study aims to examine how depression, nonproductive thoughts, and problematic internet use interact with illness perception and burden to affect psychological well-being and life satisfaction. Methods: A cross-sectional study was conducted with 207 chronically ill children aged 10–18 years. The children, aged between 10 and 18 years old, attended regular check-ups in different specialities (gastroenterology, pulmonology, onco-haematology, and paediatric rehabilitation). A cross-sectional study was carried out using psychological instruments to measure life satisfaction (SWLS), nonproductive thoughts (NPG-K), problematic internet use (PIU-Q), illness perception (PRISM) and illness burden (PRISM-D, IIRS), and depression (BDI-R). Spearman rank correlation analysis was used to explore the associations between variables. Results: Life satisfaction was negatively related to nonproductive thoughts (r = −0.28, *p* < 0.001), internet obsession (r = −0.20, *p* < 0.01), and internet neglect (r = −0.20, *p* = 0.004). Conversely, a positive correlation was found with the PRISM (r = 0.14, *p* = 0.042), suggesting that less dominance of illness detection is associated with higher life satisfaction. Depression and nonproductive thoughts showed a strong positive relationship (r = 0.49, *p* < 0.001), and depression and problematic internet use also showed significant correlations for the obsession, neglect and control subscales (r = 0.23–0.29, all *p* < 0.001). Cluster analysis identified three psychological profiles: ‘positive fighters’, ‘avoidant sufferers’, and ‘negative observers’, distinguished by differences in depression, nonproductive thoughts, illness burden, and well-being. Conclusions: The results suggest that the quality of life of children and adolescents with chronic illness is significantly affected by mental health factors, particularly depression, nonproductive thoughts and problematic internet use. Illness perception and illness-related distress also play a key role in shaping life satisfaction and overall psychosocial well-being. These findings underscore the need for targeted psychological interventions in pediatric chronic care to enhance well-being and promote adaptive coping and suggest that psychological interventions and targeted psychosocial support can significantly improve these children’s quality of life. Further research is needed to explore intervention options and to develop optimal support strategies.

## 1. Introduction

Despite growing recognition of the psychological challenges faced by children and adolescents with chronic illness, there remains a notable gap in the literature regarding how cognitive, emotional, and behavioral factors intersect to influence well-being in this population. Most previous studies have examined depression, rumination, or problematic internet use in isolation, with limited integration of these variables into a unified framework of psychological functioning. Furthermore, few studies have explored how these factors jointly relate to life satisfaction and perceived illness burden in youth with chronic conditions. This study addresses this gap by examining the complex associations among depression, nonproductive thoughts, problematic internet use, mental well-being, and life satisfaction, offering a more comprehensive understanding of the psychosocial mechanisms that influence adjustment in chronically ill youth.

The mental health of children with chronic illnesses is a particularly important research topic, as these children face not only physical challenges but also significant psychosocial burdens. Chronic illnesses are a major stressor for children and their families, and the results of a number of studies show that ongoing treatments, hospital stays and lifestyle restrictions lead to higher risks of depression, anxiety and low self-esteem [1,2]. Considering the essential domains of the bio-psycho-social model of health, health differences at the levels vary but are equally significant compared to the average propensity score. Chronic illness can affect physical health [3,4], social relationships [5], school performance [6], and quality of life [7]. One of the outcomes of this can be reduced life satisfaction due to limited activities, isolation, and lack of peer support due to illness [8]. In addition, perceptions of the illness burden are closely related to quality of life and psychological well-being [9].

Satisfaction with life is a subjective indicator of well-being that reflects how satisfying and happy an individual feels in life [10]. In children with chronic illness, this can be influenced by a number of factors, such as subjective perceived suffering of the illness, peer support, and psychological adaptability [11,12]. Studies have shown that life satisfaction is negatively related to nonproductive thoughts, i.e., the more negative, ruminative thoughts that are present, the lower the life satisfaction [13,14]. In addition, it has been shown that some aspects of problematic internet use, such as obsessive internet use and neglecting daily tasks, can also reduce quality of life [15,16].

Rumination is a repetitive, uncontrollable mindset in which the individual is constantly focused on negative events and experiences [17]. This is particularly important in children with chronic illness, as the physical and psychosocial stress of illness can significantly increase rumination [18]. Research findings show that nonproductive thoughts positively correlate with problematic internet use. This suggests that youth may turn to excessive internet use as an avoidant coping mechanism to escape ruminative thought patterns. These maladaptive strategies, while potentially offering short-term relief, may contribute to lower life satisfaction and hinder effective psychological adjustment to chronic illness [19].

Depression is a particularly high-risk condition among children with chronic illness, as coping with illness, limited physical activity and reduced social relationships can all contribute to the development of mood disorders [20,21]. Some research suggests that there is a significant negative correlation between depression and life satisfaction, i.e., the higher the level of depression, the less favourable children experience life [22]. The concept of illness burden refers to how chronic illness limits an individual’s quality of life and daily activities [23]. The subjective suffering caused by illness is positively related to depression and the impact of illness burden on intimacy, relationships and personal development [16].

Illness burden is particularly important for the emotional well-being of children and adolescents, as the perceived impact of illness can be a key determinant of adjustment [23,24]. Leventhal’s model of illness self-management is a prominent social cognitive approach that describes how patients form lay beliefs about health threats and how these beliefs influence coping [25,26]. The model emphasises cognitive and emotional beliefs, focusing on the individual perspective and recognising that the management of illness depends largely, sometimes solely, on the motivation of individuals. The model was initially intended to describe how people interpret health threats and illnesses and their coping responses; it has also been used to describe how illness beliefs are related to disease outcomes (e.g., disease progression, perceived health, functioning, psychological well-being) through the selection of coping procedures that may ultimately determine disease course and prognosis.

The research aims to explore the factors that shape the mental health of children and adolescents with chronic illnesses. We aim to better understand the psychological factors that influence the quality of life and mental well-being of children and adolescents with chronic illnesses. In the current study, we aimed to explore the following four research questions:

1. What kind of relationship can be found between satisfaction with life and its relationship with nonproductive thoughts, problematic internet use and illness perception?
2. What is the association of nonproductive thoughts with depression, problematic internet use and different aspects of illness perception?
3. What kind of connection exists between depression and psychological well-being, particularly in terms of illness-related subjective suffering and illness burden?
4. Mapping the impact of disease burden on life satisfaction, mental health and psychosocial adjustment.

Overall, we suppose that higher levels of depression, maladaptive cognitive patterns, and problematic online behaviors would be linked to reduced life satisfaction and greater perceived illness burden. In this context, the following hypotheses were formulated:

**H1.**  *Satisfaction with life is negatively related to nonproductive thoughts, problematic internet use and negative aspects of illness experience.*

**H2.**  *Higher levels of nonproductive thoughts are positively associated with depression and problematic internet use.*

**H3.**  *Higher depression scores are associated with lower satisfaction with life and a higher burden of disease.*

A further research question is what groups can be formed along the above-mentioned psychological variables and how sociodemographic characteristics shape these group memberships.

## 2. Materials and Methods

### 2.1. Sample and Data Collection

A cross-sectional study was performed. In the study, we followed the STROBE guidelines. Our study included children and one of their parents/guardians aged 10–18 years with a chronic disease who regularly attend the Paediatric Clinic’s gastroenterology, pulmonology, onco-haematology and paediatric rehabilitation departments. The inclusion of children and adolescents from diverse chronic illness groups (gastroenterology, pulmonology, oncohaematology, paediatric rehabilitation) was a deliberate methodological decision. Our primary research aim was not to study disease-specific outcomes, but rather to investigate the general psychological factors—such as depression, nonproductive thoughts, problematic internet use, and illness perception—that influence psychological well-being and life satisfaction in children with chronic illness regardless of the specific diagnosis. All included diseases were chronic, requiring long-term management and regular clinical visits, ensuring that the common lived experience of chronicity, rather than the biological specifics of each disease, was the central focus of our psychological investigation.

The research had the following inclusion criteria:

- Age between 10 and 18 years;
- Diagnosis of a chronic illness requiring long-term management and regular clinical follow-up;
- Sufficient literacy and cognitive ability to complete a psychological test battery independently (as assessed by treating clinicians);
- Parental/guardian consent and child assent to participate;

Meanwhile, the following exclusion criteria were set:

- Intellectual disability or cognitive impairment that would prevent independent questionnaire completion;
- Severe psychiatric comorbidity (e.g., active psychosis);
- Illiteracy or language barriers preventing comprehension of the test materials.

The questionnaires were administered in the personal presence of the PhD student conducting the research, each time under the supervision of the clinical psychologist professor conducting the research. Respondents were asked to complete the questionnaire on a voluntary basis. In all cases, intellectual disability and illiteracy were excluded from the study. The assumption that a 10-year-old child is able to complete a test package of this structure and length independently was also an important factor in the selection of the age group.

### 2.2. Study Instruments

We took a pre-assembled battery of tests with children and adolescents to assess the components of the personality profile of children with chronic illness that might be related to our study objective in the reviewed literature. In our self-designed Sociodemographic Questionnaire, we formulated questions regarding gender, age, type of residence, highest educational level of parents, family structure, subjectively assessed financial status, number of siblings, exact description of chronic disease(s), and achievement of independent (alone) sleep. In addition, the following psychological measures were used, that have been validated for the full age range used in the current study:

- The Satisfaction With Life Scale [27] measures well-being. It is a 5-item measure designed to assess an individual’s overall cognitive judgment of life satisfaction, distinct from the evaluation of emotional states. Participants indicate their agreement with each statement on a 7-point Likert scale, ranging from “strongly disagree” to “strongly agree”. The scale demonstrated acceptable internal consistency in the current study (Cronbach’s α = 0.74).
- The Cantril Ladder [28] also measures subjective well-being. Participants were presented with an image of a ladder numbered from 0 at the bottom to 10 at the top, symbolising the full range of possible life experiences, from the worst possible life (0) to the best possible life (10). Participants were asked to place themselves on the rung that best represented their current life situation. Scores of 4 or below indicated a state of “suffering”, whereas scores of 7 or above reflected “thriving”. Higher placements on the ladder were associated with greater perceived well-being and life satisfaction (Cronbach’s α = 0.86).
- The Nonproductive Thoughts Questionnaire (NPG-K) is a single-factor scale [13] for measuring ruminations and rumination in childhood, with scores ranging from 10 to 30. It is a unidimensional scale aimed at evaluating the tendency for rumination and perseverative negative thinking in children. It includes 10 items, each rated on a 3-point scale: 1 (“not true”), 2 (“sometimes true”), and 3 (“often true”). Higher total scores indicate a greater frequency of nonproductive thoughts. In this study, the internal reliability was strong (Cronbach’s α = 0.84).
- The Problematic Internet Use Questionnaire (PIU-Q) for adolescents, an abridged version [29], assesses attitudes and behaviors related to internet use across three subdomains. Using a 5-point Likert scale, participants express their level of agreement with various statements. The “obsession” subscale captures preoccupation with and fantasising about internet activities, as well as withdrawal symptoms when access is restricted. The “neglect” subscale evaluates the extent to which internet use interferes with daily responsibilities and essential needs. The “control disorder” subscale assesses difficulties in managing internet use. The overall internal consistency for the questionnaire was high (Cronbach’s α = 0.84).
- In the Drawing version of Pictorial Representation of Illness Self-Measure, PRISM-D [30], four subscales of the drawing test developed by the Hungarian working group of the PRISM test were considered: the distance between the yellow circle symbolising self and the circle symbolising illness (SIS), the average area of the circle representing illness 25.12 cm^2^ (IPM), the number of circles representing youth resources (KSZ), and the total area of the circles representing resources (KT) were compared.
- The Beck Depression Inventory—Shortened Scale (BDI—R) [31], the most reliable measure of depressive symptom severity. It is a 21-item self-report questionnaire that captures emotional and cognitive experiences over a recent time frame. Each item is rated on a 4-point scale from 0 to 3, resulting in a total score ranging between 0 and 84. Higher scores indicate greater severity of depressive symptoms. In this study, the BDI demonstrated good internal consistency (Cronbach’s α = 0.82).
- The Illness Intrusiveness Ratings Scale (IIRS) [32], a measure of “illness burden” as a means of assessing the impact of chronic illness and its treatment on different aspects of life is a 13-item self-report tool developed to assess the degree to which chronic illness and its treatment interfere with areas of life important to quality of life. Although initially intended for individuals coping with severe and life-threatening conditions, it is also applicable to those with less severe health issues. Respondents rate the impact of illness on various domains using a 7-point scale, from 1 (“not very much”) to 7 (“very much”), with higher scores indicating greater perceived disruption. The scale demonstrated good internal consistency in this study (Cronbach’s α = 0.86).

### 2.3. Statistical Analysis

Data were collected in an Excel sheet and data analysis was carried out with IBM SPSS 22.0 and Jamovi 2.3.28 statistical software. Descriptive statistics were generated from the questionnaire data. Non-normal distribution of the data (*p* < 0.05) was considered using non-parametric tests. Spearman rank correlation analysis was used to measure the relationships between the variables. Patient groups were created using cluster analysis (K-Means cluster, iteration: 100). We evaluated internal validation metrics, including the Silhouette Score and the Elbow Method, to determine the optimal number of clusters. The silhouette analysis revealed that the optimal cluster count was k = 3, which maximised inter-cluster separation and intra-cluster cohesion. The elbow plot showed a marked inflection at k = 3, supporting this choice.

## 3. Results

### 3.1. Sample Characteristics

Based on the compiled demographic questionnaire, a total of 207 individuals participated in the study. The gender distribution consisted of 99 boys (47.8%) and 108 girls (52.2%). The average age of the participants was 14.3 years, with a standard deviation of 2.0. In terms of residency, 123 individuals (59.49%) lived in the county seat, 21 (10.1%) in a big city, 16 (7.7%) in small towns, and 47 (22.7%) in villages. This distribution was intentionally designed to ensure a diverse representation of opinions, not solely from the county town population.

In total, 164 participants (79.1%) indicated having at least one sibling while 43 children (20.9%) reported being an only child. When considering birth order, one participant had no siblings, 123 (59.4%) were first born, 55 (26.6%) were second born, and the remaining 29 (14%) were the third or later child in their families.

The majority of the participants, specifically 83.8%, reside in their parents’ household. A smaller percentage, 9.1%, live with their mother, while an even smaller portion, 3%, live with their father. Additionally, 1% of the respondents live with a grandparent, and 3% reside with a foster parent. Concerning the living arrangements, a significant number, 80.7%, have their own room, while the remaining 19.3% do not currently possess one. Among those with a room, the majority, 78.8%, sleep in their own space. However, 11.1% share a room with their parents, and 10.1% share a room with their siblings. In terms of financial standing, the subjective evaluation provided by the participants indicates that 84% consider themselves to be living on an average income. Conversely, 13% believe they are living on a low income, while the remaining 3% claim to have a high income.

The breakdown of patients (specifically children) was as follows: 6.4% with gastroenterological, 10.1% with onco-haematological, 9.3% with pulmonological, and 15.9% with a rehabilitation-related disorder. In total, 76.64% reported that none of their immediate family members had a chronic illness.

### 3.2. Relationship Between Satisfaction with Life, Nonproductive Thoughts, Problematic Internet Use and Negative Aspects of Illness Experience

The results of the correlation analysis can be found in the Appendix A (Table A1). A significant negative correlation could be seen between Satisfaction with life and Nonproductive Thoughts (r = −0.28; *p* < 0.001), suggesting that the more nonproductive thoughts, the lower the satisfaction with life.

Problematic Internet Use Obsession (r = −0.201; *p* < 0.01) and Neglect (r = −0.201; *p* = 0.004) were found to be associated with lower life satisfaction, suggesting that Internet Use Obsession and Neglect in other life domains are associated with lower quality of life.

Satisfaction with life is significantly positively related to the PRISM circles area variable (r = 0.142; *p* = 0.042), suggesting that a higher level of life satisfaction was found to be associated with more positive illness representation.

### 3.3. Relationship Between Nonproductive Thoughts, Depression and Problematic Internet Use

Nonproductive thoughts are positively correlated with the obsession (r = 0.185; *p* = 0.008) and control subscales (r = 0.249; *p* < 0.001) of Problematic Internet Use, which may suggest that higher levels of problematic Internet use are associated with increased levels of nonproductive thoughts.

Nonproductive thoughts positively correlate with the PRISM disease circle size variable (r = 0.270; *p* < 0.001), suggesting that higher disease detection is associated with higher nonproductive thoughts.

Subjective suffering due to illness in PRISM is moderately positively associated with depression (r = 0.299; *p* < 0.001), i.e., higher subjective suffering due to illness is associated with higher levels of depression.

The PRISM disease subjective suffering is moderately positively related to the total illness burden index (r = −0.203; *p* = 0.004), as well as to illness burden relationships and personal development (r = −0.242; *p* = 0.001), intimacy (r = −0.301; *p* < 0.001) and instrument subscale (r = −0.232; *p* = 0.001), suggesting that higher disease subjective suffering is associated with higher illness burden.

### 3.4. Relationship Between Depression, Satisfaction with Life and Burden of Disease

There is a significant negative correlation between Depression and Satisfaction with life (r = −0.367; *p* < 0.001), meaning that higher levels of depression are associated with lower satisfaction with life.

There is a strong positive relationship between Depression and Nonproductive Thoughts (r = 0.491; *p* < 0.001), which may suggest that higher levels of depression correlate with higher levels of nonproductive thinking

For each subscale of Depression and Problematic Internet Use (obsession: r = 0.291; *p* < 0.001; neglect: r = 0.262; *p* < 0.001; control: r = 0.227; *p* = 0.001), i.e., higher levels of depression and problematic Internet use go hand in hand.

### 3.5. Characteristics of Patient Groups

We looked at how groups can be formed along the lines of burden of disease, well-being, nonproductive thoughts and depression levels. The results of the cluster analysis showed that the three groups were well distinguishable (Table 1). The negative observers (*n* = 22) were characterised by a high level of illness burden, more nonproductive thoughts, higher levels of depression and lower well-being. The group of avoidant sufferers (*n* = 63) was characterised by low illness burden, high levels of nonproductive thoughts and depression, and low subjective well-being. The group of positive fighters (*n* = 121) was characterised by lower disease burden and depression, fewer nonproductive thoughts and higher well-being.

In terms of gender distribution, the results of the Chi-square test show a significant difference in distribution (*p* = 0.031). In the group of avoidant sufferers, girls are over-represented in one group. Moreover, in the group of positive fighters and negative observers, boys are present in a higher (but not over-represented) proportion (Table 2).

There was also a significant difference in the proportions of cluster memberships by mother’s education (*p* = 0.005). Children of mothers with secondary education were underrepresented among avoiding sufferers and overrepresented among negative observers. In contrast, children of mothers with tertiary education were overrepresented among avoiders and underrepresented among negative contemplators. Significant distributional differences were also found for the father’s educational level (*p* = 0.015), with a marked overrepresentation of negative perceivers among children of mothers with primary education (Table 3).

No significant difference in distribution was found for family structure (*p* = 0.385, Table 4).

**Figure 1:**
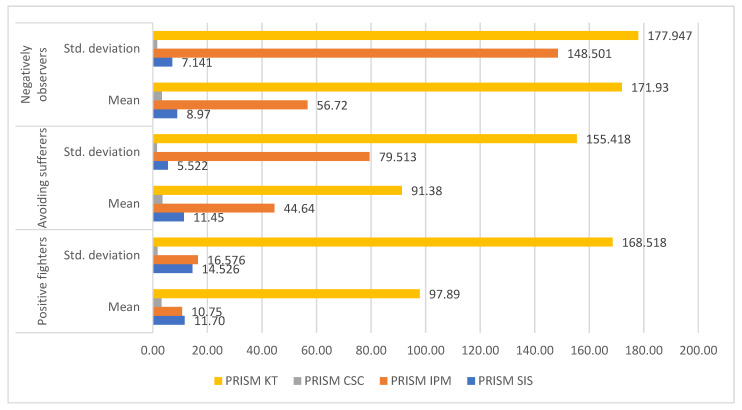
PRISM test differences between groups.

Finally, we measured each cluster’s illness representation. The Kruskal–Wallis test revealed a significant difference only in terms of the size of the illness presented (IPM) (*p* = 0.002). The cluster of negative observers had the highest IPM, followed by the avoiders and then the positive observers. There was no significant difference between the groups in SIS (*p* = 0.616), number of circles (*p* = 0.352), and size of circles (*p* = 0.280) (Figure 1).

## 4. Discussion

The aim of this study was to investigate how depression, nonproductive thoughts, problematic internet use, and illness burden are associated with psychological well-being and life satisfaction among children and adolescents with chronic illnesses. Building on the bio-psycho-social model of health, we hypothesised that higher levels of depression, maladaptive cognitive patterns, and problematic online behaviors would be linked to reduced life satisfaction and greater perceived illness burden. Our findings support these hypotheses and contribute to the growing body of literature emphasising the complex interplay between emotional, cognitive, and behavioral factors in the adjustment to chronic illness during youth. In the following discussion, we compare our results with previous research, highlight novel contributions, and outline practical implications for psychosocial interventions and future studies.

Overall, our H1 hypothesis is confirmed. Reduced satisfaction with life is associated with higher levels of nonproductive thoughts, suggesting that continuous rumination and negative thinking significantly impair subjective well-being [33]. This may be particularly important because nonproductive thoughts can increase stress reactions and hinder effective problem-solving, further exacerbating emotional distress and deterioration in quality of life [34].

Problematic Internet use (obsession and neglect) also negatively affects satisfaction with life, probably because Internet addiction makes children less likely to participate in other activities that provide positive experiences. In addition, excessive internet use may contribute to isolation in social relationships, poorer sleep quality and reduced academic performance, all of which may indirectly further reduce subjective well-being [35,36].

Satisfaction with life is positively related to the area of the PRISM circles, suggesting that a lower subjective illness burden may be associated with higher satisfaction with life. This may suggest that children and adolescents who perceive more resources in coping with illness, such as family and friends’ support or internal psychological resilience, feel less constrained by the limiting effects of illness and consequently are more satisfied with their lives [37,38,39].

Our hypothesis, H2, is partially confirmed. Nonproductive thinking is positively associated with problematic internet use. This may indicate that some children use the Internet to avoid negative thoughts, but it may also exacerbate rumination and depressive symptoms [18]. Problematic Internet use may be a maladaptive coping strategy that may alleviate distress in the short term but may increase anxiety and isolation in the long term, thus further deepening mental health problems [40,41].

The positive correlation between disease perception and nonproductive thoughts suggests that increased attention to the disease and its perceived severity may lead to rumination [33]. This may suggest that children who experience their illness as a determinant may be more prone to be trapped in a cycle of negative thoughts, which may hinder effective emotional processing and psychological adjustment [42].

The subjective suffering caused by illness is associated with depression, meaning that the more a child suffers due to the limitations of the illness, the higher the risk of developing depression [43,44]. This may be particularly important for the psychosocial development of children with chronic illness, as persistent illness can limit interaction with peers, school performance and future plans, all of which may contribute to the development of depressive symptoms [45,46].

The link between illness burden and depression is particularly strong in the areas of relationships, personal development and intimacy, meaning that for children with chronic illness, barriers to social interaction and self-actualisation can have particularly severe psychological effects [47,48]. This may also suggest that psychosocial support, such as family, friends or professional help, may be of particular importance in mitigating the psychological effects of chronic illness. Emotional support and an inclusive environment can facilitate successful coping with illness and maintenance of psychological well-being [49,50].

Our H3 hypothesis is also confirmed. Depression and nonproductive thinking are strongly positively related, confirming that negative thinking patterns play a significant role in the maintenance of depression [51]. Rumination—the repeated, uncontrollable recollection of negative thoughts—may not only contribute to the persistence of depression but may also exacerbate it by increasing emotional distress and reducing the ability to problem-solve [52] actively. This phenomenon can be particularly dangerous for developing children and adolescents, as it may affect their long-term self-esteem and coping strategies.

Our hypothesis, H4, is partially confirmed. The association between depression and problematic internet use suggests that children with depression may be more prone to escape to the Internet, mainly through obsessive use and neglect of daily activities [53]. This may result in a vicious circle where internet addiction further exacerbates depressive symptoms [35]. Spending too much time online can lead to a breakdown in real-world social relationships, a decrease in physical activity and sleep disturbances, all of which can contribute to worsening depression [54,55]. In addition, constant exposure to the Internet, especially social media use, can increase self-esteem problems and the negative effects of social comparisons, which can also deepen depressive states [56].

The cluster analysis results identified three distinct groups based on illness burden, well-being, nonproductive thoughts and depression levels. Negatively perceiving people were characterised by high illness burden, increased depression levels, more nonproductive thoughts and low well-being. Avoidant sufferers have low disease burden, high depression levels, high levels of nonproductive thinking and low subjective well-being. The largest group, the positive copers, is characterised by low disease burden, fewer nonproductive thoughts, lower depression levels and higher well-being. Gender differences show that girls are over-represented among avoiders, while boys dominate the other two groups. This may suggest that girls may be more prone to emotional avoidance or suppression, for example, in stressful situations or when anxious, while boys tend to respond more directly to problems, for example, by acknowledging negative emotions or actively managing problems [57,58]. Social norms, parenting patterns and gender roles may also influence this difference [59]. Parental education also influences cluster membership: negative talkers are more likely to be children of parents with lower education, while children of parents with higher education are more likely to be avoidant sufferers. This may suggest that parents’ educational attainment may be related to children’s problem-solving abilities and mental health. Parents with higher education may be better able to provide more supportive and proactive parenting.

In contrast, parents with lower education levels are often less able to help children deal with mental health problems, which may lead to negative attitudes [60,61,62]. There are also differences in illness perception, with negative copers perceiving the highest level of illness burden while positive copers perceive the least. This may suggest that those who perceive situations negatively tend to perceive problems more strongly, which can cause anxiety and additional stress [63]. In contrast, those who use positive coping strategies tend to perceive a lower burden of disease, as they are more optimistic and better able to manage stress and challenges [64].

The limitations of the research include the size and composition of the sample. The study sample is not representative, so the results cannot be generalised to the whole population. From a methodological point of view, it is necessary to mention that self-report questionnaires may bias the results. Moreover, the cross-sectional nature of the research does not allow for the identification of specific effects, which justifies the need to conduct longitudinal research. Moreover, we should mention that while the PIU measured aspects such as obsession, neglect, and control, the specific content of internet use (e.g., health-related searches, entertainment, peer communication) was not assessed. Identifying the nature of online activities could provide further insights into how certain types of content might exacerbate or mitigate rumination and illness perception. However, the aim of this study was to explore general problematic internet behaviours rather than content-specific patterns. Future research should include detailed assessments of the types of information adolescents seek online and how these behaviours interact with their illness-related cognitions and emotional adjustment. Additionally, further research should consider prevention and intervention to decrease adverse mental conditions and increase positive mental health by integrating alternative therapeutic [65,66] or artificial intelligence-supported methods [67,68,69].

## 5. Conclusions

This study highlights that psychological factors, such as depression, nonproductive thoughts, problematic internet use, and illness burden, play a central role in shaping the life satisfaction and well-being of children and adolescents living with chronic illnesses. Higher levels of depressive symptoms and rumination were significantly associated with lower satisfaction with life and higher illness burden. Problematic internet use also emerged as a maladaptive coping mechanism linked to decreased well-being. Cluster analysis identified three distinct psychological profiles, indicating that the interplay between mental health, cognitive patterns, and illness perception creates varying risk levels for reduced life satisfaction. These findings underline the critical importance of integrating psychological screening and targeted mental health interventions into chronic care for young patients. Future research should explore longitudinal trajectories and content-specific patterns of internet use to deepen understanding and guide intervention development.

The results of this research highlight that the mental health of children with chronic illness is influenced by several interrelated psychological factors (satisfaction with life, depression, nonproductive thoughts, problematic internet use and illness burden). These findings emphasise the importance of psychological interventions (e.g., cognitive behavioural therapy), e.g., focusing on reducing nonproductive thoughts and reducing depressive symptoms. Psychosocial support should also be highlighted to reduce the subjective suffering caused by the disease and improve quality of life. The development of conscious internet use strategies is also of paramount importance, which can help children avoid problematic use patterns. The results clearly demonstrate that targeted mental health interventions can play a key role in improving the quality of life of children with chronic conditions.

## Figures and Tables

**Table 1:** Groups that can be created based on cluster analysis.

	Clusters		
	Positive Fighters	Avoiding Sufferers	Negative Observers
IIRS_relationships	−0.03038	−0.68284	2.10529
IIRS_intimacy	−0.03066	−0.63105	1.96410
IIRS_instrument	−0.01801	−0.64992	1.95025
NPG	−0.43886	0.60906	0.66958
BDI-R	−0.46333	0.57115	0.91273
SWLS	0.50984	−0.73287	−0.72073
Cantril ladder	0.42707	−0.61695	−0.65843

**Table 2:** Gender differences in cluster membership.

		Clusters			Total
		Positive Fighters	Avoiding Sufferers	Negative Observers	
**boy**	N	62	22	14	98
	Row%	63.3%	**22.4%**	14.3%	100.0%
	Adjusted Residual	1.3	−2.4	1.6	
**Girl**	N	59	41	8	108
	Row %	54.6%	**38.0%**	7.4%	100.0%
	Adjusted Residual	−1.3	2.4	−1.6	
**Total**	N	121	63	22	206
	Row %	58.7%	30.6%	10.7%	100.0%

Note: Bolded results refer to significant over- (adj- res. > 2) or underrepresentation (adj. res < −2).

**Table 3:** Differences in cluster membership by parental educational attainment.

			Clusters			Total
			Positive Fighters	Avoiding Sufferers	Negative Observers	
father’s educational level	primary	N	7	4	4	15
		Row %	46.7%	26.7%	26.7%	100.0%
		Adjusted Residual	−1.1	−0.2	1.9	
	secondary	N	51	16	14	81
		Row %	63.0%	**19.8%**	**17.3%**	100.0%
		Adjusted Residual	0.7	−2.3	2.2	
	tertiary	N	57	35	4	96
		Row %	59.4%	**36.5%**	**4.2%**	100.0%
		Adjusted Residual	−0.1	2.4	−3.2	
Total		N	115	55	22	192
		Sor%	59.9%	28.6%	11.5%	100.0%
mother’s education	primary	N	9	3	6	18
		Row %	50.0%	16.7%	**33.3%**	100.0%
		Adjusted Residual	−0.9	−1.3	3.3	
	secondary	N	56	32	9	97
		Row %	57.7%	33.0%	9.3%	100.0%
		Adjusted Residual	−0.7	1.1	−0.6	
	tertiary	N	49	21	5	75
		Row %	65.3%	28.0%	6.7%	100.0%
		Adjusted Residual	1.2	−0.4	−1.4	
Total		N	114	56	20	190
		Row %	60.0%	29.5%	10.5%	100.0%

Note: Bolded results refer to significant over- (adj- res. > 2) or underrepresentation (adj. res < −2).

**Table 4:** Differences in cluster membership by family structure.

			Clusters			Total
			Positive Fighters	Avoiding Sufferers	Negative Observers	
coexistence	with mother and father	N	21	9	6	36
		Row %	58.3%	25.0%	16.7%	100.0%
		Adjusted Residual	−0.1	−0.8	1.3	
	only with mother/father/other family member	N	100	54	16	170
		Row %	58.8%	31.8%	9.4%	100.0%
		Adjusted Residual	0.1	0.8	−1.3	
Total		N	121	63	22	206
		Row %	58.7%	30.6%	10.7%	100.0%

## Data Availability

Data are available only upon request due to ethical restrictions. For further information, please contact the following email address: kovacs.karolina@arts.unideb.hu.

## Appendix A

**Table A1:** Correlations between psychological variables based on the results of Spearman’s rank correlation test.

		1	2	3	4	5	6	7	8	9	10	11	12	13	14
Satisfaction with life	Correlation Coefficient	1.000	−0.289 **	−0.201 **	−0.253 **	−0.121	−0.035	−0.123	0.105	0.142 *	−0.367 **	0.088	0.054	0.129	0.026
	Sig. (2-tailed)		0.000	0.004	0.000	0.083	0.617	0.082	0.134	0.042	0.000	0.207	0.442	0.064	0.705
Nonproductive thoughts	Correlation Coefficient	−0.289 **	1.000	0.185 **	0.124	0.249 **	−0.115	0.270 **	−0.055	−0.090	0.491 **	−0.063	−0.043	−0.054	−0.039
	Sig. (2-tailed)	0.000		0.008	0.077	0.000	0.103	0.000	0.433	0.204	0.000	0.365	0.536	0.439	0.577
Problematic internet use—obsession	Correlation Coefficient	−0.201 **	0.185 **	1.000	0.378 **	0.409 **	0.002	0.061	−0.147 *	−0.017	0.291 **	0.055	0.089	0.073	0.054
	Sig. (2-tailed)	0.004	0.008		0.000	0.000	0.977	0.388	0.036	0.814	0.000	0.433	0.203	0.295	0.442
Problematic internet use—neglect	Correlation Coefficient	−0.253 **	0.124	0.378 **	1.000	0.325 **	0.102	0.103	0.078	0.131	0.262 **	−0.115	−0.105	−0.127	−0.096
	Sig. (2-tailed)	0.000	0.077	0.000		0.000	0.149	0.149	0.270	0.063	0.000	0.101	0.131	0.068	0.172
Problematic internet use—control	Correlation Coefficient	−0.121	0.249 **	0.409 **	0.325 **	1.000	0.010	0.057	0.009	−0.006	0.227 **	−0.071	−0.076	−0.090	−0.072
	Sig. (2-tailed)	0.083	0.000	0.000	0.000		0.883	0.425	0.898	0.936	0.001	0.308	0.275	0.198	0.305
PRISM—subjective suffering caused by illness	Correlation Coefficient	−0.035	−0.115	0.002	0.102	0.010	1.000	0.392 **	0.208 **	0.315 **	−0.075	−0.058	−0.146 *	−0.151 *	−0.101
	Sig. (2-tailed)	0.617	0.103	0.977	0.149	0.883		0.000	0.003	0.000	0.288	0.412	0.037	0.031	0.152
PRISM—Disease circle size	Correlation Coefficient	−0.123	0.270 **	0.061	0.103	0.057	0.392 **	1.000	0.296 **	0.368 **	0.299 **	−0.203 **	−0.242 **	−0.301 **	−0.232 **
	Sig. (2-tailed)	0.082	0.000	0.388	0.149	0.425	0.000		0.000	0.000	0.000	0.004	0.001	0.000	0.001
	N	201	200	200	200	200	201	201	201	201	200	201	201	201	201
PRISM—number of rounds	Correlation Coefficient	0.105	−0.055	−0.147 *	0.078	0.009	0.208 **	0.296 **	1.000	0.469 **	−0.007	−0.009	−0.033	−0.053	0.000
	Sig. (2-tailed)	0.134	0.433	0.036	0.270	0.898	0.003	0.000		0.000	0.926	0.896	0.638	0.453	0.997
PRISM—area of circles	Correlation Coefficient	0.142 *	−0.090	−0.017	0.131	−0.006	0.315 **	0.368 **	0.469 **	1.000	−0.051	−0.043	−0.148 *	−0.151 *	−0.111
	Sig. (2-tailed)	0.042	0.204	0.814	0.063	0.936	0.000	0.000	0.000		0.472	0.538	0.035	0.032	0.113
depression	Correlation Coefficient	−0.367 **	0.491 **	0.291 **	0.262 **	0.227 **	−0.075	0.299 **	−0.007	−0.051	1.000	−0.134	−0.083	−0.196 **	−0.084
	Sig. (2-tailed)	0.000	0.000	0.000	0.000	0.001	0.288	0.000	0.926	0.472		0.055	0.234	0.005	0.230
disease burden—all	Correlation Coefficient	0.088	−0.063	0.055	−0.115	−0.071	−0.058	−0.203 **	−0.009	−0.043	−0.134	1.000	0.924 **	0.906 **	0.929 **
	Sig. (2-tailed)	0.207	0.365	0.433	0.101	0.308	0.412	0.004	0.896	0.538	0.055		0.000	0.000	0.000
disease burden—relationships and personal development	Correlation Coefficient	0.054	−0.043	0.089	−0.105	−0.076	−0.146 *	−0.242 **	−0.033	−0.148 *	−0.083	0.924 **	1.000	0.919 **	0.933 **
	Sig. (2-tailed)	0.442	0.536	0.203	0.131	0.275	0.037	0.001	0.638	0.035	0.234	0.000		0.000	0.000
disease burden—intimacy	Correlation Coefficient	0.129	−0.054	0.073	−0.127	−0.090	−0.151 *	−0.301 **	−0.053	−0.151 *	−0.196 **	0.906 **	0.919 **	1.000	0.896 **
	Sig. (2-tailed)	0.064	0.439	0.295	0.068	0.198	0.031	0.000	0.453	0.032	0.005	0.000	0.000		0.000
disease herd—device	Correlation Coefficient	0.026	−0.039	0.054	−0.096	−0.072	−0.101	−0.232 **	0.000	−0.111	−0.084	0.929 **	0.933 **	0.896 **	1.000
	Sig. (2-tailed)	0.705	0.577	0.442	0.172	0.305	0.152	0.001	0.997	0.113	0.230	0.000	0.000	0.000	

* *p* < 0.05; ** *p* < 0.01.
